# Merkel Cell Carcinoma of the Head and Neck: Recommendations for Diagnostics and Treatment

**DOI:** 10.1245/s10434-017-5993-1

**Published:** 2017-07-31

**Authors:** Urs Dietmar Achim Müller-Richter, Anja Gesierich, Alexander Christian Kübler, Stefan Hartmann, Roman Camillus Brands

**Affiliations:** 1 Department of Oral and Maxillofacial Plastic Surgery, Würzburg University Hospital, Würzburg, Germany; 2 Department of Dermatology, Venereology and Allergology, Würzburg University Hospital, Würzburg, Germany; 3 Interdisciplinary Center for Clinical Research, Würzburg University Hospital, Würzburg, Germany; 4 Comprehensive Cancer Center Mainfranken, Würzburg University Hospital, Würzburg, Germany

## Abstract

**Background:**

Merkel cell carcinoma (MCC) is a rare, aggressive tumor that often occurs in the head and neck region. Because of these features, the classifications and diagnostic and treatment regimens are frequently modified. Especially in the anatomically complex head and neck region, it is crucial to be aware of the current recommendations for diagnostics and treatment of MCC to ensure appropriate treatment. This overview aims to summarize the currently available literature.

**Methods:**

The authors reviewed the relevant literature and international guidelines for MCC from 2012 to 2017 with respect to epidemiology and prognosis, diagnostic procedures and imaging, surgery, radiation, systemic treatment, and aftercare. These results were compared with existing guidelines, some of them current, and recommendations were derived.

**Results:**

Marked developments in imaging have resulted in an increased use of functional imaging. The surgical concepts have changed regarding safety margins and the use of sentinel node biopsies. In systemic treatment, a move from conventional agents toward immuno-oncology can be observed.

**Conclusions:**

For staging, it is important to be as exact as possible using functional imaging (e.g., positron emission tomography/computed tomography scan), especially in the head and neck area with its complex lymph drainage. This often plays an especially important role in early stages of the tumor, when the resection margin can be reduced to preserve the organ. Aftercare also should include functional imaging. In an advanced, metastatic stage, immuno-oncology (PD-1, PD-L1, CTLA-4) is superior to the previous methods of systemic treatment.

Merkel cell carcinoma (MCC) is a relatively recent tumor, first described in 1972 by Cyril Toker (“trabecular carcinoma of the skin”).^1^ Generally manifested on the skin, MCC features epithelial and neuroendocrine differentiation and is rare, with an incidence of 0.13 per 100,000 (Europe) to 1.6 per 100,000 (Australia).^2^,^3^ The incidence is increasing, possibly due to better diagnostics and detection or increasing lifespans.^4^

Findings show MCC to be extremely aggressive, with high locoregional metastasis and recurrence rates and high lethality (higher than for malignant melanoma). Reported rates for deaths within 5 years range from 55% (localized tumor) to 84% (metastatic tumor).^5^

Commonly, MCC manifests on the head/neck and arms in skin exposed to sunlight. The primary manifestation of MCC is a painless, rapidly growing cutaneous, often reddish or purple nodule.^6^ Typical risk factors, besides exposure to ultraviolet (UV) radiation, are immunosuppression [human immunodeficiency virus (HIV) or organ transplants], advanced age (>50 years; median ~75 years), fair skin, and previous tumor disease.^6^ The acronym AEIOU describes the characteristics of MCC (*a*symptomatic, rapid *e*xpansion, *i*mmunosuppression, *o*lder than 50, and *U*V exposure of light skin).^7^ However, this acronym is only a starting point because these features are present in many benign and malignant skin lesions.^8^

A large follow-up study of 14,000 patients investigating stage distributions found that 50.6% of the patients had local tumor, 35.4% had nodal involvement, and 13.5% had distant metastases.^9^ Men are affected more often than women.^2^ Most patients are white (94.9%), with only 1% having dark skin. Other ethnic groups form the remaining 4.1%.^10^

## Pathogenesis

Previously, clinicians assumed that MCC originated in Merkel cells, originally described in 1875 by Friedrich Sigmund Merkel as “tactile epithelial cells”.^11^ These cells are present at the dermis–epidermis junction, form a complex with sensory neurons, and play a role in mechanoreception and afferent impulse conduction.^12^ Recent studies have questioned this theory that MCC originate in Merkel cells. The exact tumor genesis also has not been fully clarified.^13^,^14^ A multi-factorial process likely comprises immune suppression, UV damage to the skin (also virus-negative), and virus involvement.^6^ Virus-negative MCC also differs from virus-positive MCC, with a considerably higher mutation burden [e.g., p53, NOTch, neurofibromin 1 (NF1), fibroblast growth factor receptor 2 (FGFR2), phosphoinositide 3-kinase/AKT serine/threonine kinase (PI3K/AKT)], probably due to increased UV exposure.^15^^–^ 18 Immunocompromisation also promotes MCC. Organ transplants, malignant diseases, HIV, immunosuppressive drugs, and especially chronic lymphocytic leukemia can increase the risk of MCC 34–48-fold.^7^

The Merkel cell polyomavirus (MCPyV), a non-enveloped double-stranded DNA virus associated with pathogenesis, was first described by Feng et al.^19^ in 2008. The virus is present in normal ubiquitous dermal flora.^20^ The exact method of infection transmission, which occurs in childhood, is asymptomatic, and persists lifelong, has not been conclusively explained.^20^ The virus remains detectable lifelong in antibodies whose quantity correlates with the viral burden.^21^,^22^

Despite the high prevalence of MCPyV, the incidence of MCC is very low (~3 per 1,000,000).^2^,^3^ However, evidence of integrated MCPyV DNA is high (66–80%) and pathognomonic for the presence of MCC.^19^,^23^ The cap-dependent translation regulator (4E-BP1) is activated by the small tumor antigen (ST). The members of the pocket protein family [retinoblastoma protein (pRB), retinoblastoma-like protein 1 (p107), and retinoblastoma-like protein 2 (p130)] are inactivated by the large tumor antigen (LT).^20^,^24^ Both processes later activate survivin, which reduces the apoptosis rate.^24^ However, it is unclear whether the prognosis for MCPyV-positive tumors differs from that for MCPyV-negative tumors.^22^

## Prognostic Factors

The clinical factors associated with shorter survival include age older than 75 years, male gender, primary location on the lips, and tumor diameter greater than 2 cm.^25^ This corresponds with the histopathologic features of tumor extension beyond the dermis (tumor thickness), positive resection margins, and a high mitosis rate.^25^,^26^ In addition, immunosuppression and vitamin D deficiency have a negative correlation with survival.^27^,^28^ The effect of T cell infiltration and PD-L1 status has not been confirmed to date, but there may be positive correlations with an improved survival rate.^29^

Locoregional metastasis in the head and neck and distant metastasis are particularly important. Studies have shown that the proximity and absence of pathologic evaluation of locoregional lymph nodes are associated with poorer survival.^30^^–^ 32 Locoregional lymph nodes should therefore always be examined histopathologically. Metastasis and the number of lymph nodes positive for metastasis also are associated with poorer survival and sometimes with a greater tendency for locoregional recurrence.^30^^–^ 33 Therefore, a sentinel lymph node biopsy (SLNB) should always be the first step when no clinical suspicion of locoregional metastasis exists, and for positive findings, neck dissection of the drainage region should be performed.^6^ This leads to lower rates of recurrence in these regions. This does not, however, prevent distance metastases, and the presence of locoregional metastases diminishes the prognosis considerably.^34^

The sensitivity and specificity of SLNB can be increased significantly by navigated techniques.^35^ A stepwise procedure reduces postoperative complications and impairment.^36^ In addition to locoregional lymph node metastasis, the skin, central nervous system, bones, and liver are later typical sites of metastasization.^37^,^38^

## Imaging

Depending on the author and experience, for primary staging, sonography, magnetic resonance imaging (MRI), computed tomography (CT), or positron emission tomography (PET)/CT with 18-fluorodeoxyglucose (18-FDG) imaging is recommended, with no consensus for a specific method to date.^36^ Usually, CT imaging is used because one study showed evidence of its superior sensitivity and specificity over MRI imaging.^39^ Combined functional imaging such as PET/CT and PET/MRI could be advantageous for certain questions and suspected distant metastases.^40^ More distant metastases were detected in some studies when PET/CT was used in the initial examination, which led to upstaging of the patients and could be evidence supporting the use of these methods.^41^,^42^ However, a conclusive evaluation of PET/CT used for MCC is not possible. A few comparative studies of the head and neck cancer have shown relatively slight differences between diffusion-weighted MRI, PET/CT, and PET/MRI imaging in the initial staging examination.^43^^–^ 45

A chest examination is very important in the aftercare of MCC. Therefore, in addition to a clinical examination every 8–12 weeks after primary treatment, regular imaging of the head/neck and chest is recommended, which should be supplemented by occasional sonographies.^36^ Due to the significance of the first posttreatment examination after 3 months, some authors recommend PET/CT.^46^,^47^ The use of PET/CT scans with radioactive somatostatin analogs instead of 18-FDG are a new approach because these receptors are expressed by MCC.^48^,^49^ Radiotracers bound to octreotides (^68^Ga DOTATATE or ^68^Ga DOTATOC) are used.^48^ Another advantage of these tracers is that tumors are detected that can be treated using somatostatin analogs. The disadvantage is the higher background uptake in the liver, adrenal glands, pancreas, thyroid, and spleen.^50^

## Staging

The consensus guideline (8th ed) of the American Joint Committee on Cancer (AJCC) from 2016 is based on a study of more than 9000 MCCs.^9^ The classifications are presented in the new AJCC classification (Tables 1, 2) based on tumor size, type of local metastasis, or distant metastasis. Local nodal involvement must be histopathologically confirmed. If nodal metastasis is suspected, an attempt can be made to confirm it by fine-needle aspiration or core biopsy. With additional clinical or pathologic confirmation of the diagnosis, five groups with several subgroups can be formed. These subgroups have high prognostic relevance (Tables 1, 2). In the new classification, patients with unknown primary tumor are classified as stage 3a, which reflects the better clinical course of these patients. Additional tumor biologic characteristics such as those known for some other tumors (e.g., lymphangiosis carcinomatosa) have not been integrated into this staging classification to date.

**Table 1:** American Joint Committee on Cancer (AJCC) classification (8th ed): tumor–node–metastasis (TNM) classification^9^

T	X	0	Is	1	2	3	4
	Not assessable	Not present	In situ	≤2 cm	>2 and ≤5 cm	>5 cm	Invasion of bone, muscle, fascia, or cartilage

cN	X	0	1	2	3
	Not assessable (e.g., the regional lymph nodes were already removed earlier)	No clinically or radiologically detected metastasis to regional lymph nodes	Clinically detected metastasis to one or more locoregional lymph nodes	In-transit metastasis without nodal metastasis	In-transit metastasis with nodal metastasis

pN	X	0	1a(sn)	1a	1b	2	3
	Not assessable (e.g., the regional lymph nodes were already excised earlier or were not excised during treatment)	Not present	Clinically occult micrometastasis now detected by SLNB	Clinically occult metastasis to one or more locoregional lymph nodes	Clinically detected metastasis to one or more locoregional lymph nodes	In-transit metastasis without nodal metastasis	In-transit metastasis with nodal metastasis

M	0	1	1a	1b	1c
	Not present	Metastasis beyond the locoregional lymph drainage region	Metastasis to not adjacent skin, subcutaneous tissue, or distant lymph nodes	Metastasis to the lungs	Metastasis to other regions

*SLNB* sentinel lymph node biopsy

**Table 2:** American Joint Committee on Cancer (AJCC) classification (8th ed): stage aggregation^9^

Stages (clinical)		T	N	M	Stages (pathologic)		T	N	M
0		Tis	N0	M0	0		Tis	N0	M0
1		T1	pN0	M0	1		T1	pN0	M0
2	A	T2/3	pN0	M0	2	A	T2/3	pN0	M0
	B	T4	cN0	M0		B	T4	cN0	M0
3		T0–4	N1–3	M0	3	A	T1–4	N1a(sn) or N1a	M0
						B	T0	N1b	M0
						C	T1–4	N1b–3	M0
4		T0–4	Each N	M1	4		T0–4	Each N	M1

## Treatment

Because MCC disease is so rare and due to the rapid progression and metastasis, treatment of the disease is interdisciplinary.^51^ This is even more important for MCCs of the head and neck, especially the lips, because they appear to have a poorer prognosis than peripheral MCCs outside the head and neck region.^52^,^53^ The most important component of this classification is the R0 excision of the primary lesion up to the fascia with a safety margin of at least 1 cm in stage 1 disease and at least 2 cm in higher stages,^54^^–^ 56 an SLNB or lymph node excision, and depending on the histopathologic finding and the resulting staging (e.g., positive nodal involvement), adjuvant (radio)therapy (minimum, 50–55 Gy in 2 or 2.5 Gy doses).^26^,^57^,^58^

Although confirmed tumor-free margins with the respective safety margin are the most important goal in MCC treatment, this must be weighed in the head and neck against mutilation (e.g., exenteration, facial nerve). To spare as much tissue as possible in such cases, special examination techniques (e.g., Mohs surgery) can be used.^59^,^60^ However, this technique should not be used indiscriminately because it appears to promote the development of in-transit metastases.^54^,^61^ No precise statement on the ideal width of the resection margin between 1 and 2 cm can be made to date because studies have shown no differences in the recurrence-free interval.^52^ Extending the safety margin beyond 1 cm has had no prognostic advantage.^37^

Adjuvant radiation for clinical N0 neck dissection or negative SLNB and confirmed resection is disputed for MCC. Depending on the study, advantages for survival have been found, but not always.^38^,^62^^–^ 64 Adjuvant radiation is therefore routinely performed at the authors’ hospital and is always indicated for positive lymph nodes or patients with a high risk of recurrence (e.g., narrow [<1 cm] or resection margin not tumor free or evidence of lymphangitis carcinomatosa).^65^ Primary radiotherapy can be administered to inoperable patients, but the 5-year survival rates are inferior to those for patients with primary resection. Adjuvant postoperative radiation seems to increase local tumor control but has no significant impact on overall tumor-related survival.^63^,^66^ It is important, however, not to delay the start of adjuvant radiation because of narrow resection margins. Prospective studies have shown that the local resection status had no effect on local tumor control when adjuvant radiation was given.^67^ Nevertheless, R0 resection should always be the goal if possible.^56^

For a clinically N0 neck, SLNB is important because regional metastasis affects the prognosis and decisions for adjuvant treatment. No consensus exists regarding the indication for radiation in the case of negative SLNB. The limitations of SLNB in the head and neck must be taken into consideration.^68^ The indication for radiation may, however, be established for larger tumors or tumors with features of aggressive growth (e.g., lymphangitis carcinomatosa). Similar to the findings in head and neck tumors, regional (micro)metastases were detected in the early stages of MCC using SLNB for up to one third of patients.^25^,^32^,^33^ Depending on the technique used, the success rates for SLNB can differ significantly between the head/neck and the remainder of the body due to the more difficult anatomic situation and more complex lymph drainage pathways. This disadvantage can be reduced by using navigated methods.^35^

The SLNB should be performed before tumor resection because otherwise, alterations can arise in the lymph territory, making the procedure more difficult. If metastasis is found in the SLNB, the probability of recurrence increases from 39 to 56%.^33^

For reliable detection of micrometastases, more sophisticated histopathologic techniques (slice thicknesses, incision lines, immunohistochemistry) are needed.^69^,^70^ Immunohistochemistry staining is indicated especially to differentiate the MCC (also primary tumor) from metastases, mainly metastases of a small cell lung cancer, melanoma, or lymphoma. For this, cytokeratin 20 (CK20–MCC), thyroid transcription factor-1 (TTF-1–SCLC), melan-A/MART-1 (melanoma), and the leukocyte common antigen (LCA–lymphoma) are used. Other useful markers are chromogranin A, neuron-specific enolase, and synaptophysin.^25^,^71^ Depending on the patient’s general condition and operability, primary radiation of the tumor and the lymph drainage territory may be considered.

Conventional chemotherapy plays a less important role than other forms of treatment (e.g., surgery or radiation) for primarily operable MCC due to the short duration of remission and high toxicity. No reliable data proving an advantage for either overall or recurrence-free survival are available.^72^^–^ 75 Chemotherapy generally is considered only for palliative care or for stage 4 disease with inoperable distant metastases. The substances used are anthracyclines, platinum (cisplatin or carboplatin), and/or etoposide and/or topotecan.^76^^–^ 78 No established systemic treatment based on validated evidence has been determined to date.

Several studies have investigated targeted therapy for MCC with limited success. Various tyrosine kinase inhibitors, for example against PDGFR or c-kit, are used (pazopanib, imatinib).^79^,^80^ Testing of a survivin inhibitor is still in the preclinical phase and has shown some success.^24^,^81^ The presence of PI3K mutations appears to be indicative of a more aggressive course and could also be a target.^82^ Inducing an immune response through immune modulators such as imiquimod, which binds to TLR7, has shown some success.^83^ The expression of somatostatin receptors also allows the possibility of peptide receptor radionuclide therapy, for example with ^177^Lu-DOTATATE, which studies are investigating.^84^ No valid data are available for these various active substances.

The use of PD-1 and PD-L1 inhibitors also is very promising, and the data are superior to the data of other systemic treatments. Expression of PD-1 and PD-1 ligand (sometimes high) has been proven in both virus-positive and virus-negative MCC.^15^,^18^ The expression strength appears to increase with higher tumor stages.^85^ This indicates an immunogenic reaction to the tumor and is supported by the fact that the quantity of CD8^+^ T cells in the tumor correlates with the probability of survival.^29^,^86^,^87^ The use of checkpoint inhibitors (PD-1, PD-L1, CTLA-4) therefore may be useful for MCC as well.

To date, the PD-1 antibody pembrolizumab and the PD-L1 antibody avelumab have been investigated as first- or second-line treatment in advanced tumor stages (3 and 4).^88^,^89^ The response rates have been 32–56%. Complete remission has been found in approximately 10–15% and partial remission in 20–40% of cases.^88^,^89^ The rates are thus comparable with those for other tumors (e.g., head and neck cancers). Success has been achieved for both virus-positive and virus-negative tumors. This is encouraging and supports suggestions for the use of these therapies as first-line treatment in advanced tumor stages, especially because the side effects of these substances generally are controlled easily.

The anti-PD-L1 antibody avelumab is Food and Drug Administration-approved in the US for metastatic MCC and is in the approval process in Europe. Currently, other ongoing studies are investigating checkpoint inhibitors for MCC (ipilimumab, tremelimumab, durvalumab, nivolumab).

Based on these data, clinicians are discussing systemic treatment with active substances from this substance class for advanced local tumors or higher metastasis rates.

## Aftercare

No aftercare protocols exist for MCC. Because 90% of all MCC recurrences happen within 24 months (median 8 months), the first 2 years are especially important for aftercare.^37^,^72^ During this period, a whole-body skin examination, including the locoregional lymph nodes, and ultrasound of the locoregional lymph nodes are recommended every 8–12 weeks. This interval can later be extended to 6–12 months. These examinations should be supported by tomographic imaging depending on risk. Especially during the first 2 years of aftercare, annual PET/CT scans can help to detect metastases.^42^

## Conclusions

MCC remains an interdisciplinary challenge, in part because it is so rare. In imaging diagnostics, it appears that functional imaging with PET tracers has become the established diagnostic method. Treatment still consists of resection with a safety margin of at least 1 cm for stage 1 or 2 cm disease in later stages if possible. The lymph drainage territory should be assessed with SLNB or neck dissection if metastases are suspected. Depending on the risk profile or the presence of metastases, adjuvant radiation is indicated for local tumor control. For advanced-stage disease or recurrences, checkpoint inhibitors (PD-1, PD-L1, CTLA-4) could improve current treatment options.
